# The Pumilio-domain protein PUF6 contributes to SIDER2 retroposon-mediated mRNA decay in *Leishmania*

**DOI:** 10.1261/rna.062950.117

**Published:** 2017-12

**Authors:** Hiva Azizi, Carole Dumas, Barbara Papadopoulou

**Affiliations:** 1 Research Center in Infectious Diseases, CHU de Quebec Research Center-Laval University, Quebec, QC, G1V 4G2 Canada; 2 Department of Microbiology, Infectious Diseases and Immunology, Faculty of Medicine, Laval University, Quebec, QC, G1V 0A6 Canada

**Keywords:** *Leishmania*, SIDER2 retroposons, MS2 coat protein tethering system, Pumilio proteins, PUF6, mRNA decay

## Abstract

*Leishmania* and other trypanosomatid protozoa lack control at the level of transcription initiation and regulate gene expression exclusively post-transcriptionally. We have reported previously that *Leishmania* harbors a unique class of short interspersed degenerate retroposons (SIDERs) that are predominantly located within 3′UTRs and play a major role in post-transcriptional control. We have shown that members of the SIDER2 subfamily initiate mRNA decay through endonucleolytic cleavage within the second conserved 79-nt signature sequence of SIDER2 retroposons. Here, we have developed an optimized MS2 coat protein tethering system to capture *trans*-acting factor(s) regulating SIDER2-mediated mRNA decay. Tethering of the MS2 coat protein to a reporter RNA harboring two MS2 stem–loop aptamers and the cognate SIDER2-containing 3′UTR in combination with immunoprecipitation and mass spectrometry analysis led to the identification of RNA-binding proteins with known functions in mRNA decay. Among the candidate SIDER2-interacting proteins that were individually tethered to a SIDER2 reporter RNA, the Pumilio-domain protein PUF6 was shown to enhance degradation and reduce transcript half-life. Furthermore, we showed that PUF6 binds to SIDER2 sequences that include the regulatory 79-nt signature motif, hence contributing to the mRNA decay process. Consistent with a role of PUF6 in SIDER2-mediated decay, genetic inactivation of PUF6 resulted in increased accumulation and higher stability of endogenous SIDER2-bearing transcripts. Overall, these studies provide new insights into regulated mRNA decay pathways in *Leishmania* controlled by SIDER2 retroposons and propose a broader role for PUF proteins in mRNA decay within the eukaryotic kingdom.

## INTRODUCTION

*Leishmania* spp. are unicellular eukaryotic pathogens causing a wide spectrum of pathologies in humans ranging from cutaneous to visceral infections (Desjeux 2004). *Leishmania* has a digenetic life cycle alternating between extracellular promastigotes in the insect vector and amastigotes inside the phagolysosome of mammalian macrophages where they replicate and cause disease (Bates and Rogers 2004). Similarly to other trypanosomatids, the *Leishmania* genome is organized in long polycistronic units (Myler et al. 1999; Ivens et al. 2005). In the absence of transcriptional control by RNA polymerase II in trypanosomatids, polycistronic units are transcribed in a constitutive manner and further processed to mature mRNAs through a coordinated 5′-*trans*-splicing and 3′-polyadenylation cleavage reactions (Papadopoulou et al. 2008; Michaeli 2011). In addition, gene expression has been shown to change dramatically throughout the complex life cycles of these parasites (Haile and Papadopoulou 2007; Rochette et al. 2008; Nilsson et al. 2010; Siegel et al. 2010). Regulation of mRNA decay rates through interactions of RNA-binding proteins (RBPs) with *cis*-acting sequences in 3′UTRs of trypanosomatid transcripts is central in determining the fate of mRNAs and thus fine-tuning developmental gene expression (McNicoll et al. 2005; Bringaud et al. 2007; Haile and Papadopoulou 2007; Haile et al. 2008; Müller et al. 2010b; Kramer 2012; Clayton 2013). Several RBPs, such as zinc finger proteins, Alba-domain proteins, and Pumilio (PUF) proteins, have been found to regulate mRNA stability in trypanosomatids (Clayton 2013; Dupé et al. 2014).

The genomes of trypanosomatids contain a large repertoire of RBPs, few of which have been characterized to interact with specific sequences in 3′UTRs. In *Trypanosoma cruzi*, UBP1 binds to an AU-rich RNA instability element (ARE) in the 3′UTR of small mucin mRNAs and promotes RNA destabilization (D'Orso and Frasch 2001). In *T. brucei,* PUF9 binds to a consensus sequence in 3′UTR of transcripts whose function is important in temporal coordination of the kinetoplast and nuclear replication during late S-phase (Archer et al. 2009). The *T. brucei* ZC3H11 binds to and stabilizes mRNAs encoding chaperones required for protein refolding following heat shock (Droll et al. 2013). DRBD3, a protein containing two RRM domains, plays a role both in splicing and mRNA stability in *T. brucei* (Estévez 2008; Das et al. 2012). Also, DRBD13 has been demonstrated to negatively regulate mRNAs encoding for cell membrane-associated proteins via interaction with AREs of the target transcripts (Jha et al. 2015). More recently, we showed that the *Leishmania infantum* Alba3 protein can stabilize the developmentally regulated *amastin* transcripts specifically in the amastigote stage upon binding to a U-rich element in the 3′UTR (Dupé et al. 2014).

In *Leishmania*, we have previously identified a large family of extinct retroposon elements termed short interspersed degenerate retroposons (SIDERs) (>2000 copies per genome), predominantly located within 3′UTRs (Bringaud et al. 2007, 2008; Smith et al. 2009). Members of the two major SIDER subfamilies were shown to regulate mRNA turnover in a stage- and species-specific manner (SIDER2 subfamily) (Bringaud et al. 2007; Rochette et al. 2008; Müller and Papadopoulou 2010; Müller et al. 2010a,b) or mRNA translation (SIDER1 subfamily) (Boucher et al. 2002; McNicoll et al. 2005), and possibly to form RNA regulons (Ouellette and Papadopoulou 2009; Smith et al. 2009). We showed that degradation of SIDER2-bearing transcripts is initiated by endonucleolytic cleavage (Müller et al. 2010b; Papadopoulou et al. 2015) as opposed to the default process in eukaryotes, which begins with poly(A) shortening by deadenylases followed by 5′ cap removal and 5′–3′ degradation by the XRN1 exoribonuclease or 3′–5′ degradation by the exosome (Schoenberg and Maquat 2012). Cleavage was mapped at AU/CU dinucleotides within the second tandem 79-nt hallmark sequence (signature II) (Müller et al. 2010a; Azizi et al. 2017b) that is conserved at the 5′-end of all SIDER2 retroposons but not SIDER1 (Bringaud et al. 2007; Smith et al. 2009). More recently, we showed that SIDER2-harboring mRNAs have to associate with translating ribosomes in order to be degraded (Azizi et al. 2017a), suggesting that the putative endoribonuclease and associated mRNA decay factors have to be recruited to the translation apparatus.

In this study, we followed up on the mechanism of SIDER2-mediated mRNA decay by searching for *trans*-acting factors regulating this process. To this end, we have developed a bipartite MS2 coat protein pull-down system to identify RNA-binding protein candidates bound specifically to a SIDER2-containing reporter RNA. In vivo UV-crosslinking and immunoprecipitation combined with LC–MS/MS studies allowed us to identify several candidate proteins bound to a SIDER2-harboring 3′UTR but not to a 3′UTR lacking SIDER2. Among the candidate RNA-binding proteins with known functions in mRNA decay, the Pumilio-family member (PUF6) was shown to enhance degradation and reduce transcript half-life upon tethering to SIDER2 regulatory sequences. Furthermore, we showed that genetic inactivation of PUF6 leads to an increased stability of endogenous SIDER2-bearing transcripts, supporting a role of PUF6 protein in the SIDER2-mediated mRNA decay process.

## RESULTS

### Development of an improved MS2 coat protein tethering system for identifying *trans*-acting factors regulating SIDER2 retroposon-mediated mRNA decay in *Leishmania*

**FIGURE 1.**
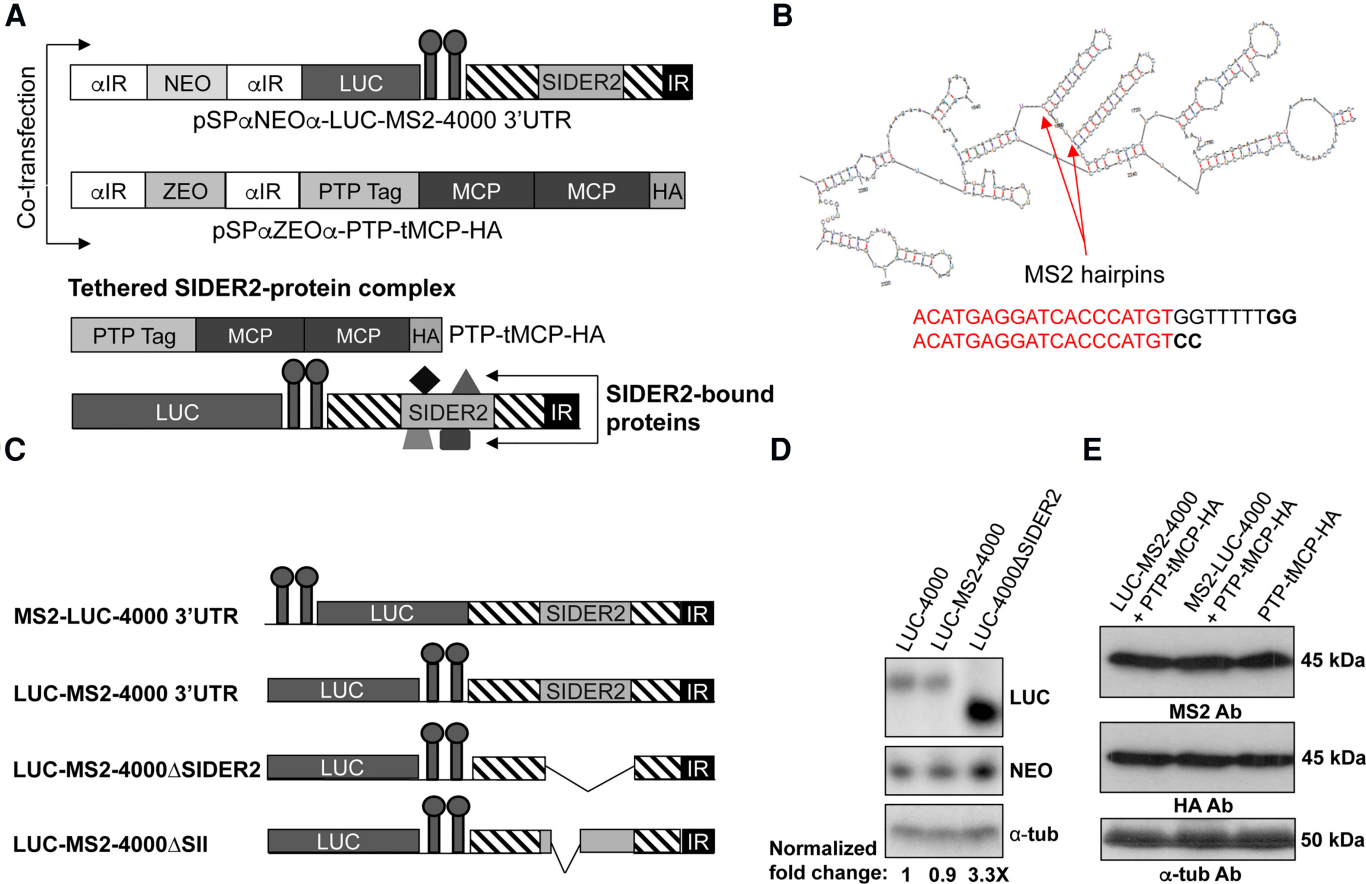
Schematic representation of the MS2 coat protein tethering system optimized and adapted for use in *Leishmania*. (*A*) This bipartite system consists of two vectors cotransfected into *L. infantum*: the first (pSPαZEOα-PTP-tMCP-HA) expressing two tandem (t) copies of the bacteriophage MS2 coat protein (MCP) with a PTP-tag at the N terminus and a HA-tag at the C terminus; and the second (pSPαNEOα-LUC-MS2-4000 3′UTR) a luciferase (*LUC*) reporter gene whose expression is driven by a SIDER2-harboring 3′UTR (3′UTR of the *L. infantum* LinJ.36.4000 transcript harboring a SIDER2 element) and two MS2 hairpins. αIR, intergenic region of the α-tubulin gene allowing *trans*-splicing and polyadenylation; NEO, neomycin phosphotransferase gene; ZEO, zeocin gene for plasmid selection following transfection. Two MS2 hairpins and a tandem MCP dimer were used to optimize binding affinity of MCP to MS2 RNA. Binding of MCP to the MS2 hairpins also tethers proteins specifically interacting with the SIDER2 RNA that can be identified by coimmunoprecipitation and LC–MS/MS studies. (*B*) mFold mRNA secondary structure prediction of the LUC-MS2-4000 3′UTR. The two forming MS2 hairpin structures in silico are indicated by arrows, and the two MS2 binding sites (ACATGAGGATCACCCATGT) are indicated in red. (*C*) Schematic representation of the LUC-MS2-4000 3′UTR constructs generated and used in this study. The full-length LinJ.36.4000 3′UTR or truncated 3′UTR lacking either SIDER2 (ΔSIDER2) or the second hallmark signature sequence (79-nt) of SIDER2 retroposons (ΔSII) were cloned downstream from the *LUC* reporter gene. (*D*) Northern blot hybridization of total RNA from parasites expressing chimeric *LUC* transcripts regulated by the LinJ36.4000 3′UTR or the truncated 3′UTR. Blots were hybridized with a LUC-specific probe, and the α-tubulin and NEO probes were used as loading controls. Normalized *LUC* mRNA levels relative to the full-length LUC-4000 3′UTR (control) are shown *below* the blot. Hybridization intensity signals were quantified using the ImageQuant 5.2 software, and *LUC* mRNA values were normalized to the α-tubulin and *NEO* mRNAs. Shown here is one representative experiment out of two independent experiments yielding similar results. (*E*) Western blot analysis on total lysates from cotransfectants and the PTP-tMCP-HA single transfectant using anti-HA and anti-MCP antibodies to detect the MCP protein. The anti-α-tub antibody was used as loading control.

So far, the λN peptide has been used successfully to tether RBPs to a particular RNA of interest in the related *Trypanosoma* species (Delhi et al. 2011; Wurst et al. 2012; Droll et al. 2013; Jha et al. 2014; Singh et al. 2014). Here, we have developed an improved MS2 coat protein tethering system adapted for use in *Leishmania* to attach RNA-binding proteins (RBPs) specifically to a reporter RNA. A schematic diagram describing this system is shown in Figure 1A. The bacteriophage MS2/R17, MS2 coat protein (MCP) recognizes and binds to a specific stem–loop aptamer termed MS2 stem–loop (MS2 SL) of the replicase open reading frame to suppress its translation (Bernardi and Spahr 1972). This observation was later applied successfully to many fields of RNA biology, including mRNA localization, capturing of ribonucleoprotein complexes, mRNA translation, and decay (Keryer-Bibens et al. 2008; Buxbaum et al. 2015). We used the mFold RNA secondary structure prediction software to determine the best flanking sequences required to form a MS2 stem–loop structure. By testing different flanking sequence combinations, we were able to find the most suitable sequence allowing the tandem MS2 hairpin RNA to form in silico (Fig. 1B). Figure 1C displays four *LUC*-MS2 reporter constructs containing or not a SIDER2 element that were used in this study. In *Saccharomyces cerevisiae,* it was recently reported that binding of MCP to MS2 SL, inserted into 3′UTR of the genes encoding for QCR8 and PGK1RNA, blocked XRN1 5′–3′ exoribonuclease activity and led to the accumulation of 3′ mRNA fragments containing MS2 SLs (Garcia and Parker 2015). To ascertain that the insertion of two MS2 hairpin structures downstream from the *LUC* gene and its interaction with MCP does not alter decay rates of the *LUC*-SIDER2 reporter transcripts, we compared MS2-LUC-4000 3′UTR along with the non-MS2 RNA, LUC-4000 3′UTR by Northern blot hybridization (Fig. 1D). As we have reported previously, SIDER2 retroposon promotes rapid RNA decay, whereas deletion of SIDER2 from the 3′UTR blocks degradation and causes accumulation of the *LUC* reporter mRNA (Fig. 1D; Bringaud et al. 2007; Müller et al. 2010b). Here, we show that addition of two MS2 hairpins in the reporter RNA and binding to MCP does not alter degradation by SIDER2 (Fig. 1D). Furthermore, we improved the MS2 pull-down system by optimizing expression levels of MCP. A single copy of MCP was not expressed sufficiently when transfected into *L. infantum* (data not shown). In the absence of an effective inducible protein expression system in *L. infantum*, we decided to express MCP episomally to obtain higher levels of expression and consequently sufficient binding to the MS2 SL RNA. Therefore, we generated a tandem MCP construct (tMCP) as MCP binds the hairpin as preformed dimers, thus recruiting two copies of the fused RBP of interest to the tethering site. Bound coat protein dimers interact cooperatively with one another when tandem arrays of hairpins are present (Johansson et al. 1997). The use of tandem MCP has already been shown in mRNA localization studies to improve the signal when fused to GFP (Wu et al. 2012). Western blot using an anti-HA or an anti-MCP antibody demonstrated a suitable expression of the tMCP protein in our transfectants (Fig. 1E), favoring the coat protein–ligand binding in vivo.

### Identification of RNA-binding proteins interacting with a SIDER2 reporter RNA using the MS2 coat protein tethering system

The MS2 coat protein tethering system is a robust in vivo approach to study the functional properties of RBPs by attracting them to any RNA of interest (Keryer-Bibens et al. 2008). To identify RBPs bound to the SIDER2-harboring 3′UTR of the LinJ.36.4000 transcript, we carried out coimmunoprecipitation (co-IP) studies against the MCP protein combined with LC/MS-MS analysis. For these studies, we used *L. infantum* parasites coexpressing the PTP-tMCP-HA protein with either the LUC-MS2-4000 3′UTR (harbors the full-length SIDER2-containing 3′UTR of LinJ.36.4000 transcript with two MS2 hairpins placed downstream from the *LUC* reporter gene) or the MS2-LUC-4000 3′UTR (the two MS2 binding sites were placed upstream of the *LUC* reporter gene) (Fig. 1A,C). Placing the MS2 binding sites both upstream and downstream, the *LUC* reporter permits us to eliminate the possibility of occupational effect on SIDER2-interacting proteins imposed by MCP when it binds to MS2 RNA. UV-crosslinking followed by co-IP experiments against the PTP-tMCP-HA protein using an anti-HA antibody coupled to magnetic beads and mass spectrometry analysis revealed a number of candidate proteins, tethered to SIDER2 RNA (Table 1). The presence of PTP-tMCP-HA protein prior and after co-IP studies was verified by Western blotting (Supplemental Fig. S1). Experiments were done with both *L. infantum* promastigotes and axenic amastigotes, but only the results with promastigotes are shown here. A control with a non-SIDER2 3′UTR (LinJ.39.3990; LUC-MS2-3990 3′UTR) was also used to assess binding specificity (not shown). Only candidate proteins tethered to the LUC-MS2-4000 3′UTR mRNA but not to LUC-MS2 or LUC-MS2-3990 3′UTR (e.g., two negative controls) were considered for further analysis. These include the XRN 5′–3′ exoribonuclease, the deadenylase complex CCR4-NOT1 and NOT5 proteins, an ATP-dependent RNA helicase (Hel 3150), a lupus La protein homolog, the Pumilio-family member PUF6, an RNA-binding protein (RBP-0610), and one hypothetical protein (LinJ.29.2040) with no predicted RBP domain(s) (Table 1). NOT1 was excluded from our study as we failed to amplify the corresponding ORF (∼7 kb) for cloning purposes. Similarly, NOT5 was excluded from further investigation as we were unable to detect the fusion protein by Western blotting (see Supplemental Fig. S2) and the transfected cells experienced some growth problems.

**TABLE 1.**
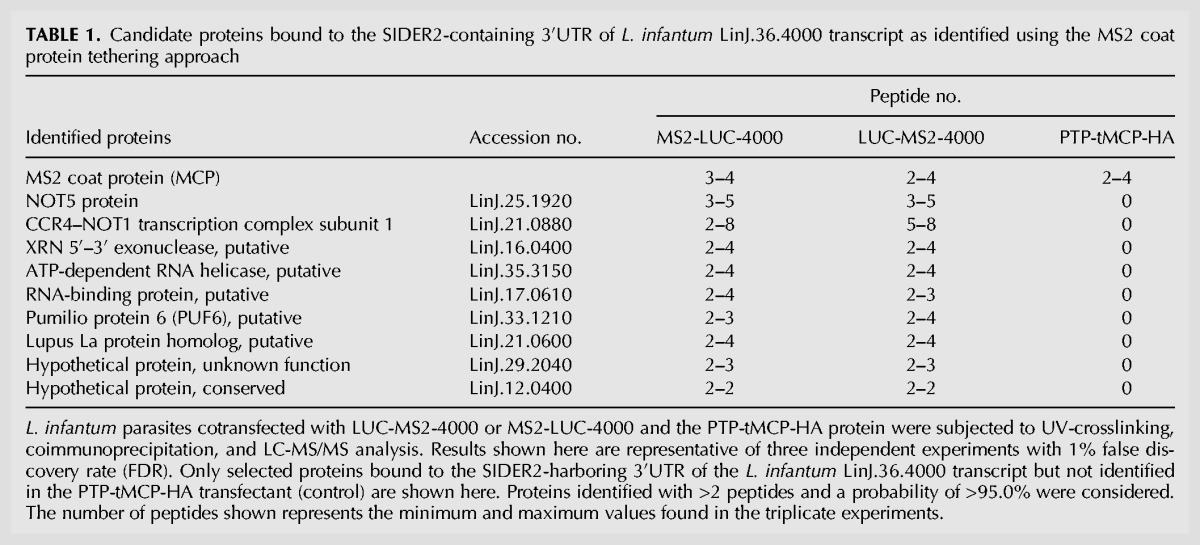
Candidate proteins bound to the SIDER2-containing 3′UTR of *L. infantum* LinJ.36.4000 transcript as identified using the MS2 coat protein tethering approach

### The Pumilio-domain protein PUF6 causes the highest mRNA destabilizing effect among the candidate SIDER2-interacting proteins once tethered to a SIDER2-harboring reporter RNA

**FIGURE 2.**
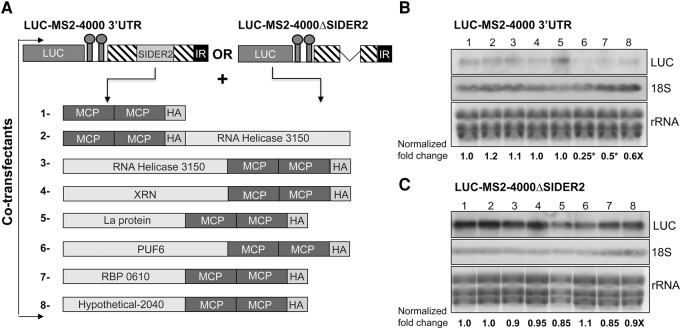
Candidate proteins tethered to a SIDER2-containing 3′UTR or to a truncated 3′UTR lacking SIDER2. (*A*) Schematic overview of the constructs made to tether candidate proteins fused with the tandem tMCP-HA to LUC-MS2-4000 3′UTR or LUC-MS2-4000ΔSIDER2 RNAs. Northern blot hybridization of total RNA isolated from *L. infantum* parasites coexpressing the candidate proteins in (*A*) and the *LUC* reporter chimeric construct with either the full-length 3′UTR of LinJ36.4000 (*B*) or a truncated 3′UTR lacking SIDER2 (*C*). The blot was hybridized with a *LUC*-specific probe to assess mRNA levels upon tethering of the different candidate proteins. The 18S rRNA probe and ethidium bromide staining were used as controls for RNA loading. Hybridization intensity signals were quantified by the ImageQuant 5.2 software and *LUC* transcript abundance was normalized to the 18S rRNA hybridization signal and also to the *LUC*-plasmid copy number in each tMCP-HA transfectant (see Supplemental Fig. S3). Normalized values indicated *below* the blot are relative to the control strain coexpressing the LUC-MS2-4000 3′UTR or the LUC-MS2-4000ΔSIDER2 and tMCP-HA (lane *1*). Shown here is one representative experiment out of three independent experiments yielding similar results. An asterisk indicates the most important differences in *LUC* mRNA levels.

To assess the role candidate SIDER2-interacting proteins may play on *LUC*-SIDER2 transcript stability, we fused mostly at the C terminus, each of these proteins with the tMCP-HA protein and coexpressed them into *L. infantum* LUC-MS2-4000 3′UTR or LUC-MS2-4000ΔSIDER2 recombinant strains (Fig. 2A) and proceeded with tethering assays on stably cotransfected cell lines. The expression of the different tMCP-fused candidate proteins was verified by Western blot analysis (Supplemental Fig. S2). No significant variation in the levels of tethered protein expression was observed between LUC-MS2-4000 3′UTR and LUC-MS2-4000ΔSIDER2 expressing parasites (Supplemental Fig. S2C). LUC-MS2-4000 plasmid copy-number variation among the different cotransfectants was determined by Southern blot hybridization (Supplemental Fig. S3). The use of episomal vectors was our best possible choice at the time as it was technically challenging to delete the entire SIDER2 sequence or the 79-nt signature II from a given 3′UTR within the genomic locus by homologous recombination. Steady-state levels of *LUC* mRNA in the different transfectants coexpressing the candidate proteins tethered to the LUC-MS2-4000 SIDER2-bearing reporter RNA were detected by Northern blot hybridization and normalized to the 18S rRNA signal (RNA loading variation) as well as to the LUC-MS2-4000 or LUC-MS2-4000ΔSIDER2 plasmid copy number (slight variations were observed between different transfectants; see Supplemental Figs. S3, S4). Normalization of the Northern blot data indicated that among the six candidate proteins investigated here, tethering of PUF6 to the LUC-MS2-4000 mRNA led to the highest effect on RNA fate, with a fourfold decrease in *LUC* mRNA levels (Fig. 2B). No destabilizing effect was observed upon tethering of PUF6 to the LUC-MS2-4000ΔSIDER2 mRNA (Fig. 2C), indicating that PUF6 has to bind sequences within SIDER2. Two other proteins, RBP-0610 and the hypothetical protein 2040, once tethered to the LUC-MS2-4000 mRNA, resulted in a 2.0-fold and 1.66-fold decrease in *LUC* mRNA levels, respectively (Fig. 2B). In this study, however, we focused on PUF6 protein, primarily due to its highest destabilizing effect on the *LUC*-SIDER2 RNA.

### Tethering PUF6 protein to a SIDER2-harboring reporter RNA enhances degradation through binding to SIDER2 regulatory sequences

**FIGURE 3.**
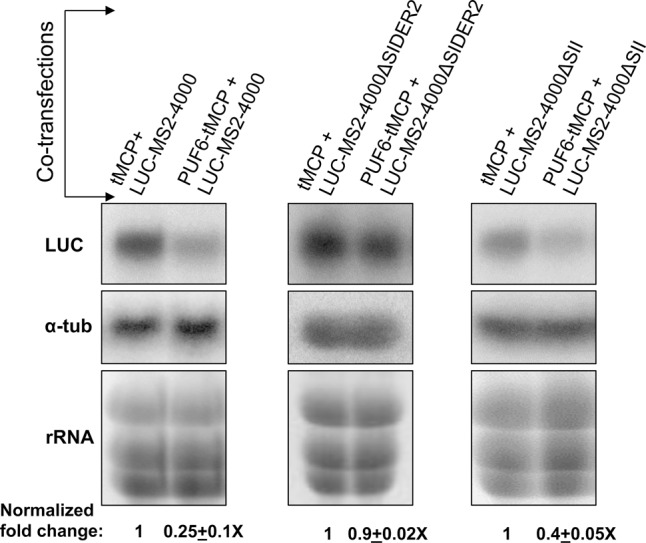
Tethering of the Pumilio 6 protein (PUF6) to a SIDER2-harboring 3′UTR enhances mRNA decay rates. Northern blot analysis of total RNA isolated from *L. infantum* coexpressing the tMCP-HA control protein or the PUF6-tMCP-HA with LUC-MS2-4000 3′UTR (*left* panel), LUC-MS2-4000ΔSIDER2 (*middle* panel), or LUC-MS2-4000ΔSII (lacking only the signature II sequence) (*right* panel). The blots were hybridized with a *LUC*-specific probe to evaluate *LUC* mRNA decay rates upon tethering of the control protein tMCP-HA or the PUF6-tMCP-HA. The α-tubulin probe was used as loading control. Fold changes of *LUC* mRNA levels upon PUF6 tethering relative to the tMCP-HA control shown *below* the blots were normalized with α-tub hybridization signals for RNA loading as well as plasmid copy numbers (see Supplemental Figs. S3–S5 for copy-number variations between plasmids in the different transfectants). Shown here is one representative experiment out of two independent experiments yielding similar results.

To determine whether PUF6 binds to the second tandem 79-nt SIDER2 signature sequence (SII), shown to be the target of endonucleolytic cleavage (Müller et al. 2010b; Azizi et al. 2017b), we generated another recombinant parasite cell line coexpressing the PUF6-tMCP protein together with the LUC-MS2-4000ΔSII mRNA. This enables us to compare the results of PUF6 tethering to the LUC-MS2-4000ΔSII with PUF6 tethering to LUC-MS2-4000 3′UTR RNAs by Northern blot hybridization. Cotransfectants with the tMCP-HA were also used as controls for comparative studies. *LUC* mRNA fold-differences were determined following normalization of the hybridization *LUC* signal intensities to the α-tubulin signal (RNA loading control) as well as to the plasmid copy number (Supplemental Figs. S3, S5). Normalized *LUC* mRNA values indicated that PUF6 confers a fourfold decrease in LUC-MS2-4000 3′UTR mRNA levels when compared to the control tMCP-HA protein tethered to the same RNA (Fig. 3, left panel). However, PUF6 did not alter *LUC* mRNA levels when tethered to a reporter transcript lacking SIDER2 (LUC-MS2-4000ΔSIDER2) (Fig. 3, middle panel), as also shown in Figure 2C. Interestingly, we found that once tethered to the LUC-MS2-4000ΔSII RNA, PUF6 does decrease *LUC* mRNA levels by 2.5-fold (Fig. 3A, right panel) as compared to fourfold when tethered to the full-length SIDER2-harboring 3′UTR (Fig. 3, left panel). Altogether, these data suggest that PUF6 binds sequences (or RNA structure) within SIDER2, including signature II, but not exclusively, to accelerate mRNA decay.

**FIGURE 4.**
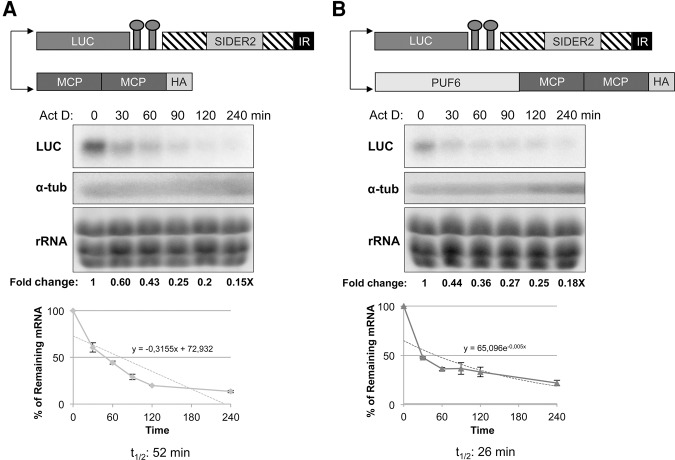
Tethering of PUF6 protein to a SIDER2-harboring 3′UTR leads to an increased mRNA destabilization. (*A*) Schematic drawing of the vectors coexpressed in *L. infantum* and used to tether tMCP-HA to the LUC-MS2-4000 3′UTR (*top* panel). Northern blot analysis of actinomycin D (ActD)-treated parasites to assess changes in *LUC* transcript stability upon tMCP tethering (control). ActD was added at the concentration of 10 µg/mL, parasites were harvested at indicated time points, and total RNA was isolated for Northern blotting (*middle* panel). Blots were hybridized with LUC or α-tubulin (loading control) probes. The numbers indicated *below* the blots represent the fold difference in *LUC* mRNA expression levels relatively to the ActD T0. These values were used to calculate the half-life of the *LUC* mRNA upon tethering of the tMCP control (*lower* panel). Due to the biphasic nature of SIDER2 mRNA degradation (Müller et al. 2010b), values corresponding to each time point were plotted in linear values of percentage of the remaining mRNA. (*B*) Schematic of the constructs used to tether PUF6 to the LUC-MS2-4000 3′UTR (*top*). Northern blot analysis of ActD-treated parasites as in *A* hybridized with LUC or α-tubulin probes to determine the half-life of the *LUC* transcript upon PUF6 tethering (*middle*). Half-lives (*t*_1/2_) were estimated when the percentage of remaining mRNA after time zero (T0) reached 50% mRNA abundance (*lower* panel). Standard deviations are from three independent experiments.

We showed that artificial binding of PUF6 to SIDER2 sequences accelerates mRNA decay (Figs. 2, 3). Further, we investigated if decreased mRNA levels upon PUF6 tethering are due to an increased destabilization. To address this question, we measured *LUC* mRNA half-lives upon tethering of PUF6 to the reporter RNA and compare them with the MCP tethering control. *LUC* mRNA stability was assessed by Northern blotting on total RNA treated prior with actinomycin D (ActD) (Fig. 4). A time course ActD treatment showed that tethering of PUF6 to the LUC-MS2-4000 3′UTR decreased mRNA stability by twofold (*t*_1/2_: 26 min) in comparison to the control experiment using the tMCP protein (*t*_1/2_: 52 min) (Fig. 4A,B). It is worth noticing that although tethering of PUF6 to the LUC-MS2-4000 3′UTR promotes a fourfold decrease in mRNA levels (Figs. 2B, 3), only a twofold destabilization was observed (Fig. 4). This may be due to the constitutive and uncontrollable tethering of PUF6 to SIDER2 mRNA. To determine half-lives of SIDER2-harboring transcripts, as shown on the *y*-axis of the charts (Fig. 4, lower panels), we decided not to use a logarithmic scale for the percentage of remaining mRNA as being used commonly because of our previous observations that SIDER2-mediated mRNA decay occurs in a biphasic manner: a rapid endonucleolytic cleavage followed by further degradation of the cleavage products via the action of exoribonucleases and deadenylases (Müller et al. 2010b).

**FIGURE 5.**
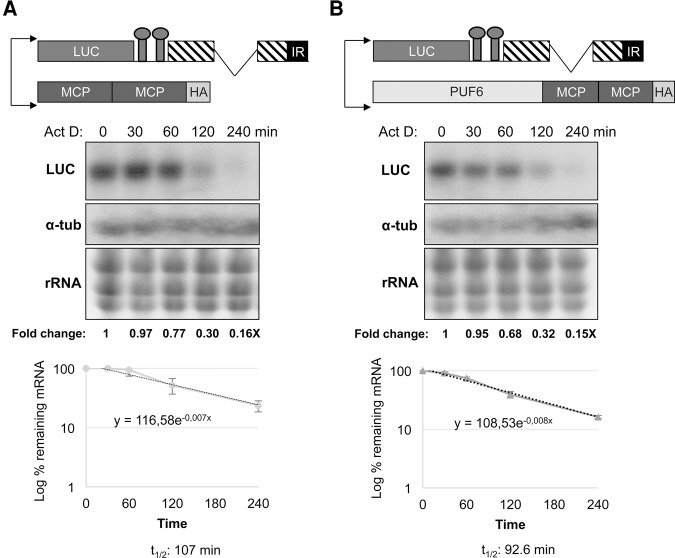
Tethering of PUF6 protein to a truncated 3′UTR lacking SIDER2 does not alter RNA stability. (*A*) Schematic drawing of the vectors coexpressed in *L. infantum* and used as a control (*top* panel). Northern blot analysis of ActD-treated parasites hybridized with LUC or α-tubulin probes (*middle*). The graph in the *lower* panel shows the half-life of the *LUC* transcript upon tethering of the control tMCP protein. (*B*) Schematic drawing of the constructs used to tether PUF6 to the LUC-4000ΔSIDER2 (*top* panel). Northern blot analysis of ActD-treated parasites as in *A* hybridized with LUC or α-tubulin probes to determine the half-life of the *LUC* transcript upon PUF6 tethering (*middle* panel). The numbers indicated *below* the blots represent the fold difference in expression ratios compared to the T0 value and were used in the half-life charts. The graph shows the half-life of the *LUC* transcript upon PUF6 tethering (*lower* panel). In the absence of the SIDER2 element, mRNA decay follows a conventional pattern. The *y*-axis referring to the percentage of remaining mRNA was set to log scale. Standard deviations are from three independent experiments.

To assess whether the PUF6-mediated destabilizing effect involves the SIDER2 element, we carried out ActD treatment on parasites coexpressing the PUF6-tMCP protein together with the LUC-MS2-4000ΔSIDER2 plasmid. As depicted in Figure 5, tethering PUF6 to a 3′UTR lacking SIDER2 did not alter the *LUC* mRNA half-life (*t*_1/2_: 92.6 min) as compared to the MCP tethering (*t*_1/2_: 107 min) (Fig. 5A,B), hence corroborating the steady-state levels of LUC-MS2-4000ΔSIDER2 mRNA (Fig. 2C). The fact that tethering of PUF6 to a SIDER2-containing reporter RNA enhances degradation supports that PUF6, through binding to SIDER2 sequences, contributes to SIDER2-mediated decay.

### Genetic inactivation of PUF6 results in increased stabilization of SIDER2-containing endogenous transcripts

**FIGURE 6.**
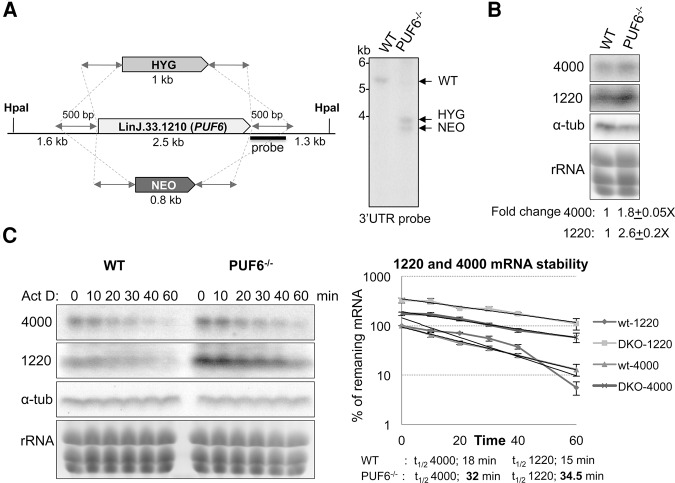
Genetic inactivation of *PUF6* leads to increased accumulation and stability of SIDER2-containing endogenous transcripts. (*A*) Schematic drawing of *PUF6* gene replacement in *L. infantum* by the neomycin phosphotransferase (*NEO*) and hygromycin B (*HYG*) expression targeting cassettes through homologous recombination (*left* panel). Southern blot hybridization of *L. infantum* wild-type (WT) and PUF6^−/−^ knockout genomic DNA digested with HpaI. The blot was hybridized with a 500-bp probe at the 3′ flanking sequence of the *PUF6* gene that detects a 5.4-kb fragment in the WT strain and 3.9- and 3.7-kb fragments in the PUF6^−/−^ knockout corresponding to the HYG and NEO gene replacements. (*B*) Northern blotting of total RNA isolated from *L. infantum* WT and PUF6^−/−^. The blots were hybridized with specific probes recognizing the *L. infantum* SIDER2-bearing transcripts, LinJ.36.4000 (4000) and LinJ.08.1220 (1220). The α-tubulin probe was used as RNA loading control. Ethidium bromide staining was also included to visualize rRNA as an additional loading control. Fold changes of 4000 and 1220 endogenous transcripts in the PUF6^−/−^ knockout relative to the WT are shown here *below* the blots and were calculated after normalization against the loading controls. Standard deviations are the result of three independent experiments. (*C*) Stability of SIDER2-harboring LinJ.36.4000 and LinJ.08.1220 transcripts was compared between the WT and PUF6^−/−^ strains following a time course treatment with the RNA Pol II transcription inhibitor ActD. Parasites were collected at indicated time points, and total RNA was extracted and subjected to Northern blotting. Blots were hybridized with specific probes against LinJ.36.4000 and LinJ.08.1220 as well as the α-tubulin gene (RNA loading control) (*left* panel). Graphical display of the half-lives of *4000* and *1220* transcripts in WT and PUF6^−/−^ (*right* panel). Half-lives (*t*_1/2_) were estimated when the percentage of the remaining mRNA after time zero (T0) reached 50% of mRNA abundance.

To investigate further the role PUF6 plays in SIDER2-mediated mRNA decay, we generated a *L. infantum* PUF6 null mutant strain. Both *PUF6* alleles were successfully replaced sequentially with the hygromycin (*HYG*) and neomycin phosphotransferase (*NEO*) resistance genes, as confirmed by Southern blot hybridization (Fig. 6A), indicating that the *PUF6* gene is not essential for *Leishmania* promastigote growth. Next, we evaluated the effect of *PUF6* gene inactivation on SIDER2-harboring LinJ.36.4000 and LinJ.08.1220 transcript expression levels by Northern blot hybridization. Both transcripts have been studied extensively in our laboratory (Bringaud et al. 2007; Müller et al. 2010a,b). Interestingly, we observed an increased accumulation of both *4000* and *1220* transcripts by 1.8-fold and 2.6-fold, respectively, in the PUF6^−/−^ knockout (Fig. 6B), which further confirms that PUF6 plays a role in SIDER2-mediated decay.

To examine changes in *4000* and *1220* mRNA stability in the absence of PUF6, we treated *L. infantum* wild-type and PUF6^−/−^ strains with ActD at indicated time points, and total RNA extracted from these strains was subjected to Northern blot hybridization. Interestingly, half-lives of *4000* and *1220* transcripts were increased by at least twofold in the absence of PUF6 (from 18 min in WT to 32 min in PUF6^−/−^ for *4000*, and from 15 min in WT to 34.5 min in PUF6^−/−^ for *1220*) (Fig. 6C). These results corroborate our findings that PUF6 contributes to SIDER2-mediated mRNA decay. In another attempt to validate the effect of PUF6 depletion on the stability of *4000* and *1220* transcripts, we treated parasites with cycloheximide (CHX) to inhibit translation elongation and then analyzed total RNA extracted from those cells at various time points post-treatment by Northern blotting. The aim of this experiment was to compare accumulation rates of SIDER2-harboring transcripts between wild-type and PUF6^−/−^ parasites under conditions of global translation inhibition where de novo-synthesis of mRNA decay factors, including PUF6, could be affected. Interestingly, accumulation of both *4000* and *1220* transcripts increased by approximately 2.5- to 3.5-fold on average, after 4–6 h of CHX treatment in PUF6^−/−^ parasites compared to the wild-type strain (Supplemental Fig. S6), suggesting that PUF6 is required for optimal decay of SIDER2-bearing transcripts.

## DISCUSSION

“Tethering” approaches have been used successfully to isolate proteins that activate mRNA turnover in human cells (Clement and Lykke-Andersen 2008; Park et al. 2013; Yokoshi et al. 2014) but also in parasites (Erben et al. 2014). Here, we report for the first time in trypanosomatid protozoa, the use of the MS2 coat protein tethering system to capture RNA-binding proteins (RBPs) regulating mRNA decay. We have optimized this system for use in *Leishmania,* which combined with immunoprecipitation and mass spectrometry analysis led us to the identification of *trans*-acting factors bound specifically to SIDER2 retroposon elements and regulating SIDER2-mediated mRNA decay*.* Among the several candidate RNA-binding proteins with known functions in mRNA decay that were tethered to a SIDER2 reporter RNA, the Pumilio-domain protein PUF6 was shown to play a role in the SIDER2-mediated mRNA decay process.

We provide several lines of evidence supporting a functional role of PUF6 in SIDER2-mediated mRNA decay. We show that tethering of PUF6 to a SIDER2-harboring 3′UTR exacerbates RNA degradation but has no effect on an RNA lacking SIDER2. Accordingly, genetic inactivation of PUF6 leads to an increased accumulation and higher stability of *Leishmania* transcripts harboring a SIDER2 element in their 3′UTR. The effect of PUF6 on SIDER2-containing mRNA destabilization is the result of specific binding to sequences within SIDER2 that include, but not exclusively, the second conserved 79-nt signature sequence (signature II) of SIDER2 retroposons, which is the target of endonucleolytic cleavage (Müller et al. 2010b; Azizi et al. 2017b). Indeed, tethering of PUF6 to a SIDER2 element lacking signature II decreases RNA stability albeit to a lesser extent than tethering to the full-length SIDER2 RNA. We have shown recently that SIDER2-mediated mRNA decay is coupled to translation (Azizi et al. 2017a), and our findings here that inhibition of global protein synthesis by cycloheximide increases the accumulation of SIDER2-harboring *Leishmania* transcripts by at least two- to threefold in the PUF6^−/−^ knockout in comparison to the wild-type strain, suggest that PUF6 contributes to the decay mechanism while associated with SIDER2-bearing mRNAs on translating ribosomes.

The Pumilio proteins are well-known RNA-binding proteins in most eukaryotic lineages. This family has expanded in trypanosomatids with 10 members in *Leishmania* (Luu et al. 2006), whereas only five members were found in the yeast *Saccharomyces cerevisiae* and two in humans. In many organisms such as *S. cerevisiae, Drosophila melanogaster*, *C. elegans*, and *H. sapiens,* several Pumilio repeats within PUF proteins share the UGUR (R represents a purine) recognition sequence flanked by downstream or upstream sequences often unique to each PUF protein (Wickens et al. 2002; Miller et al. 2008; Ulbricht and Olivas 2008). In all these cases, PUF proteins comprise eight sequence repeats (called PUM repeats) and flanking N- and C-terminal regions (Zhang et al. 1997), out of which repeats 5–8 recognize the UGUR tetranucleotide motif. However, similarly to the yeast Puf1p and Puf2p homologs, the *Leishmania* PUF6 is predicted to encompass 6 PUM repeats (Supplemental Fig. S7), and this suggests that binding via a consensus sequence may be dispensable (Ulbricht and Olivas 2008). At the molecular level, PUF proteins promote translational repression and/or mRNA degradation first by interacting specifically with *cis*-elements in the 3′UTR of their target mRNAs and through complex interactions with protein cofactors and the translation and degradation regulatory components (Wang et al. 2002; Cheong and Hall 2006; Lublin and Evans 2007; Ulbricht and Olivas 2008; Chritton and Wickens 2010; Cooke et al. 2011; Miller and Olivas 2011; Quenault et al. 2011).

There is increasing evidence in the literature that members of the PUF family play an important role in mRNA decay. Indeed, members of the PUF family bind to 3′UTRs of various eukaryotic mRNAs and enhance turnover, acting combinatorially with the CCR4–NOT deadenylase complex (Quenault et al. 2011). In humans, PUF proteins, PUM1 and PUM2, recruit the CCR4–NOT complex to stimulate mRNA decay and repress translation (Van Etten et al. 2012). In *S. cerevisiae,* recruitment of CCR4p, the catalytic subunit of the CCR4–POP2–NOT deadenylase complex, by PUF3p, PUF4p or PUF5p, results in mRNA deadenylation (Goldstrohm et al. 2006, 2007; Hook et al. 2007; Lee et al. 2010). A similar mechanism has been reported in *Drosophila, C. elegans*, and humans, where recruitment of the deadenylase complex POP2p subunit by PUF proteins stimulates deadenylation of target mRNAs (Goldstrohm et al. 2006; Kadyrova et al. 2007; Suh et al. 2009). Here, MS2 protein tethering to a SIDER2-containing reporter RNA led also to the identification of CCR4–NOT1 and NOT5 deadenylases. However, our previous data support a model for SIDER2-mediated decay, which is deadenylation-independent (Müller et al. 2010b). Therefore, these deadenylases may be recruited to a SIDER2 reporter RNA through their association with PUF6, which specifically binds sequences within SIDER2. In yeast, it has been shown that PUF4p and PUF5p bind to the *HO* mRNA 3′UTR, repressing translation, triggering deadenylation and thus mRNA decay, possibly through their association with the decapping activator DCP1p and the DHH1p helicase (Goldstrohm et al. 2006, 2007; Hook et al. 2007). A similar decay mechanism has been proposed in the related trypanosomatid, *Trypanosoma cruzi,* where PUF6 binds to a subset of mRNAs and its overexpression results in the down-regulation of target transcripts in epimastigotes, possibly through its association with the TcDHH1 helicase (Dallagiovanna et al. 2008). In *Leishmania*, however, overexpression of PUF6 did not affect steady-state levels of SIDER2-containing transcripts (Supplemental Fig. S8), suggesting that for PUF6 to enhance destabilization of SIDER2 transcripts it has to be artificially tethered to these RNAs. This could imply that PUF6 binds with higher specificity to SIDER2 target mRNAs. Although coimmunoprecipitation experiments against a PUF6-HA protein were not very optimal due to proteolytic degradation of PUF6 under nondenaturing conditions, mass spectrometry studies identified DHH1 as one of the PUF6-interacting proteins together with another decay protein, the Dis3-like ribonuclease, an exoribonuclease associated with the human exosome (Staals et al. 2010) (data not shown). Additional preliminary experiments using the BioID method (Roux et al. 2012) as an alternative approach to coimmunoprecipitation, have also led to the identification of the Dis3-like ribonuclease as a PUF6-interacting factor (data not shown). Thus, PUF6 might possibly enhance decay of SIDER2-containing transcripts by facilitating the recruitment of exoribonucleases. Moreover, the fact that PUF6 accelerates mRNA decay only once tethered to SIDER2 sequences, including the second conserved 79-nt signature of SIDER2 retroposons shown previously to be central to endonucleolytic cleavage and degradation (Müller et al. 2010b; Azizi et al. 2017b), supports the possibility that PUF6 facilitates recognition of SIDER2 by the endoribonuclease.

In summary, this study led to the identification of Pumilio-domain protein PUF6 as the first *trans*-acting factor shown to interact with SIDER2 regulatory sequences and to accelerate mRNA decay. SIDER2-mediated mRNA decay is initiated through endonucleolytic cleavage(s) within the second conserved 79-nt signature sequence at the 5′end of all SIDER2 retroposons, followed by 5′–3′ and 3′–5′ degradation of mRNA ends by exoribonucleases (Müller et al. 2010b). Thus, multiple factors are expected to form the SIDER2-specific decay complex. The identification of PUF6 as one of the contributing factors to SIDER2-mediated mRNA decay would permit the isolation of other components of the decay complex and shed more light onto this complex mechanism of regulated mRNA decay in parasitic protozoa.

## MATERIALS AND METHODS

### *Leishmania* culture and transfections

*Leishmania infantum* MHOM/MA/67/ITMAP-263 (Sereno et al. 2001) promastigotes were cultured in SDM-79 medium supplemented with 10% FCS (Wisent) and 5 µg/mL hemin at 25°C. On average, 10–20 µg plasmid DNA were transfected into *Leishmania* promastigotes by electroporation as previously described (Papadopoulou et al. 1992). To generate stable transfectant cell lines, parasites were selected with 25 µg/mL G418 (Sigma-Aldrich), 10 mg/mL zeocin (Life Technologies), or hygromycin B (Sigma-Aldrich) at 1 mg/mL.

### Tethering constructs

The parent plasmid pSP72-YNEOαIR described elsewhere (Wu et al. 2000) was used to generate the LUC-MS2-3′UTR constructs. pSP72-YNEOα-LUC-MS2-4000 3′UTR (Y stands for a 92-bp polypyrimidine stretch [Papadopoulou et al. 1994]; α, for the intergenic region of the α-tubulin gene; NEO for the neomycin phosphotransferase gene; and LUC for the luciferase gene) was constructed by PCR amplification of the LinJ.36.4000 3′UTR (Phusion Taq polymerase, Thermo Scientific) using primers described in Supplemental Table S1 and subsequently cloned downstream from the *LUC* gene. The two MS2 hairpins (see Fig. 1B) were placed at the beginning of the 4000 3′UTR via PCR amplification using the forward primer containing the MS2 sequences. To generate LUC-MS2-4000ΔSIDER2, an overlapping fusion PCR approach was used. A similar strategy was used to create LUC-MS2-4000ΔSII lacking only the second 79-nt signature sequence in SIDER2. To construct plasmid pSP72αNEOαMS2-LUC-4000 3′UTR, the MS2-LUC fragment was PCR-amplified and cloned via BamHI–HindIII sites into vector pSP72αNEOα (Papadopoulou et al. 1994). Next, the 3′UTR of LinJ.36.4000 was amplified and cloned into HindIII downstream from the *LUC* gene. To create the MCP-PTP fusion plasmid, the MS2 coat protein (MCP) tagged with a PTP epitope was first amplified from plasmid pLEW100-PTP-MCP (a generous gift from Dr. Schimanski, Bern, Switzerland) and cloned into XbaI–HindIII sites of pSP72αZEOα (Richard et al. 2004). Then, the second *MCP*-encoding gene was PCR-amplified and cloned in the HindIII site of pSP72αZEOαPTP-MCP to obtain pSP72αZEOαPTP-tMCP harboring two tandem MCP copies. To generate pSP72αZEOα-tMCP, the tandem MCP from the PTP-tMCP plasmid was amplified and cloned into the XbaI site of pSP72αZEOα. Genes encoding for candidate proteins interacting with SIDER2 identified using the MS2 tethering system (see Table 1) were cloned upstream of the tMCP-HA construct. *LUC* chimeric transcripts and tMCP-fusion genes are all properly 5′-*trans*-spliced and processed by the *Leishmania enriettii* α-tubulin intergenic region (αIR). In order to make a PUF6 (LinJ.33.1210) null mutant in *L. infantum,* two targeting cassettes comprising either the hygromycin (HYG) or the neomycin (NEO) phosphotransferase genes, flanked by 500 bp DNA fragments at 5′ and 3′ regions of the *PUF6* gene, were generated by overlapping PCR. The nucleotide sequence of all primers used to make the above constructs is indicated in Supplemental Table S1.

### DNA, RNA, and protein manipulations

DNA extractions were carried out using DNAzol (Life Technologies). Plasmid copy number was estimated by Southern blot hybridization analysis. Approximately 10 µg of DNA from each transfectant digested with NdeI was hybridized with a LinJ.36.4000 3′UTR (1 kb) radiolabeled probe. Hybridization intensity signals from plasmid and genomic DNA were measured by a PhosphorImager. A ratio of the signal obtained from the plasmid DNA versus the genomic DNA was used to determine the plasmid copy number in each transfectant cell line. Total RNA from parasites was isolated by TRIzol (Life Technologies) according to manufacturer's instructions and resolved on 1% agarose formaldehyde gels. Northern blots were carried out following standard procedures (Sambrook and Russell 2001). Radioactive DNA probes corresponding to the *LUC* ORF or to the LinJ.36.4000 3′UTR were synthesized using Klenow fragment DNA polymerase I (New England Biolabs) in the presence of [α^−32^P] dCTP (PerkinElmer) and random oligonucleotides (NEB) and used in Northern or Southern blots. Western blots were performed from total *L. infantum* lysates equivalent to 2 × 10^6^ parasites in 2× Laemmli buffer. HA-tagged proteins were detected using a mouse monoclonal anti-HA tag antibody (Abmgood). The MCP was detected using an anti-MCP rabbit polyclonal antibody (EMD Millipore). Loading control was assessed by rehybridizing the same membrane with a mouse anti-α tubulin antibody (Sigma-Aldrich) or a rabbit anti-NEO antibody (EMD Millipore). Anti-mouse HRP-conjugated, anti-rabbit HRP-conjugated, or anti-goat HRP-conjugated antibodies were used as secondary antibodies. The blots were visualized by chemoluminescence with an ECL+ Western Blotting Detection Kit (GE Healthcare).

### mRNA half-lives and protein synthesis inhibition

To evaluate the stability of SIDER2-harboring mRNAs expressed as part of episomal vectors or from the genomic locus, we treated mid-log phase *L. infantum* promastigotes with 10 µg/mL of actinomycin D (ActD; Gibco-Life technologies) and 2.5 µg/mL sinefungin (Abcam) to arrest de novo transcription and pre-mRNA *trans*-splicing, respectively. Sinefungin was added 5 min prior to ActD (Li et al. 2006; Haile et al. 2008). Total RNA was isolated at desired time points and analyzed by Northern blotting. To inhibit global protein synthesis, mid-log phase promastigotes were incubated with 10 µg/mL cycloheximide (Sigma-Aldrich), and at various time points, parasites were collected, total RNA isolated and analyzed by Northern blot hybridization. Following transfer, membranes were exposed to a Phosphorimager, and signal intensity was measured using the ImageQuant 5.2 software.

### Immunoprecipitation and mass spectrometry analysis

Frozen parasite pellets were immediately resuspended in the lysis buffer comprising 25 mM Tris–HCl pH 7.4, 100 mM NaCl, 1.5 mM MgCl2, 1 mM EDTA, 0.5% NP40, 5% glycerol, and 1 mM PMSF supplemented with protease inhibitors (Roche). Lysis was completed by 20–30 strokes of a Dounce homogenizer while on ice. Cell debris and insoluble material were separated by 30 min centrifugation at 10,000*g* at 4°C. Supernatants were then incubated with Protein G magnetic beads (Thermo Scientific) for 30 min at 4°C in order to reduce nonspecific binding and to eliminate protein binding to the beads. Clear supernatants were further incubated with Protein G anti-HA magnetic beads at 4°C for 4 h on a gentle rotator. Beads were then washed by TBS-0.05% Tween (Sigma-Aldrich) three times (30 sec each) by gentle agitation and subjected to LC–MS/MS analysis as previously described (Padmanabhan et al. 2016). Immunoprecipitation experiments were done on cotransfected parasites as above except that prior to freezing the parasites in liquid nitrogen, they were exposed to 400 mJ/cm^2^ UV irradiation on a Stratalinker 2400 UV crosslinker in PBS medium and then immediately harvested and snap-frozen.

## SUPPLEMENTAL MATERIAL

Supplemental material is available for this article.

## Supplementary Material

Supplemental Material
