# Supplementary material for: The Pumilio-domain protein PUF6 contributes to SIDER2 retroposon-mediated mRNA decay in Leishmania
Source: RNA. 2017 Dec;23(12):1874–85. doi: 10.1261/rna.062950.117 (PMC5689007; doi:10.1261/rna.062950.117)
Supplement: Supplemental Material [file supp_23_12_1874__index.html]

The Pumilio-domain protein PUF6 contributes to SIDER2 retroposon-mediated mRNA decay in Leishmania — Supplemental Material 

# The Pumilio-domain protein PUF6 contributes to SIDER2 retroposon-mediated mRNA decay in *Leishmania*

## Supplemental Material

- Supplemental\_Fig\_S1.tif
- Supplemental\_Fig\_S2.tif
- Supplemental\_Fig\_S3.tif
- Supplemental\_Fig\_S4.tif
- Supplemental\_Fig\_S5.tif
- Supplemental\_Fig\_S6.tif
- Supplemental\_Fig\_S7.tif
- Supplemental\_Fig\_S8.tif
- Supplemental\_Information.docx
