# Supplementary figures and images for: The Pumilio-domain protein PUF6 contributes to SIDER2 retroposon-mediated mRNA decay in Leishmania
Source: RNA. 2017 Dec;23(12):1874–85. doi: 10.1261/rna.062950.117 (PMC5689007; doi:10.1261/rna.062950.117)

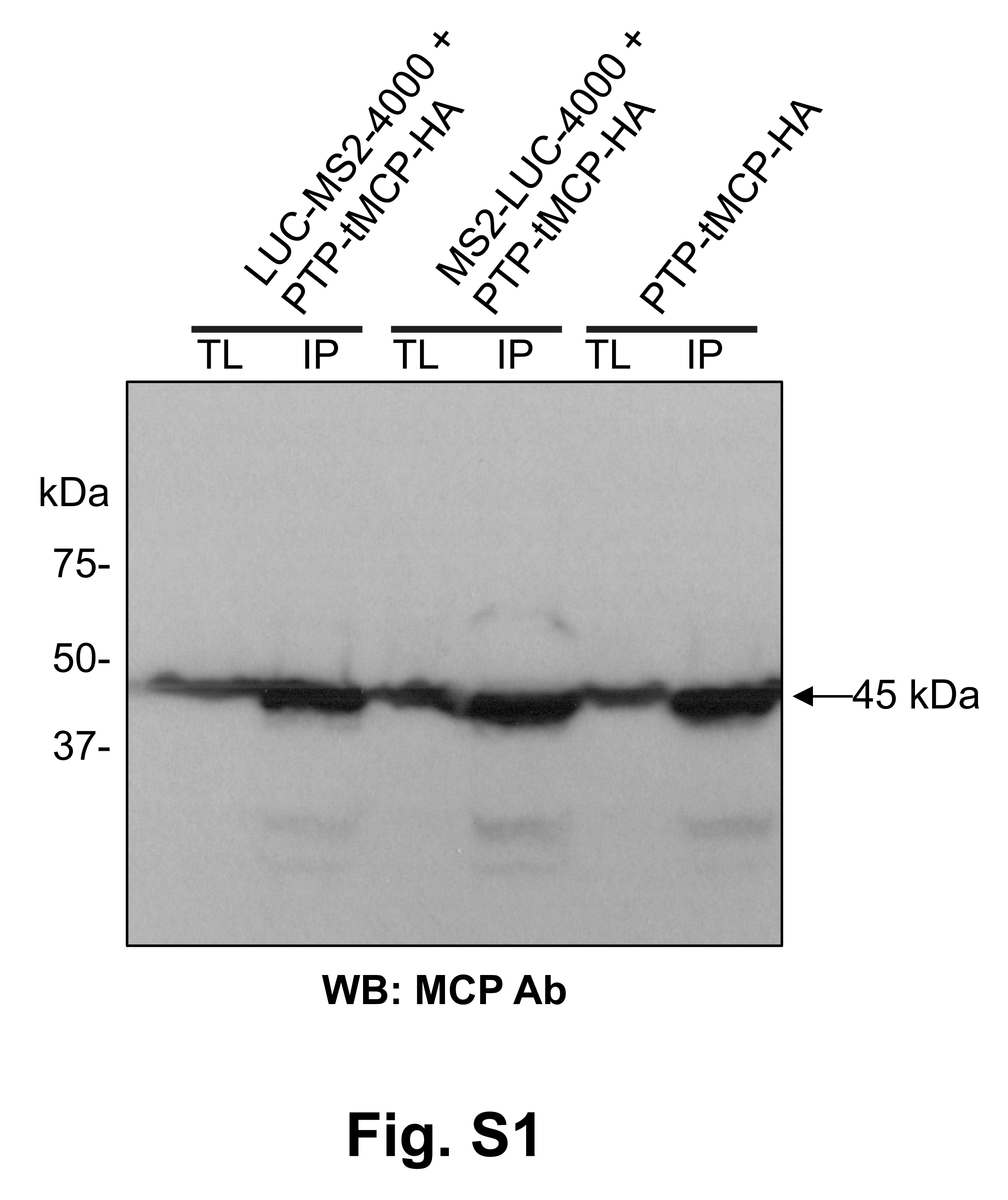

Supplement: Supplemental Material [file supp_062950.117_Supplemental_Fig_S1.tif]

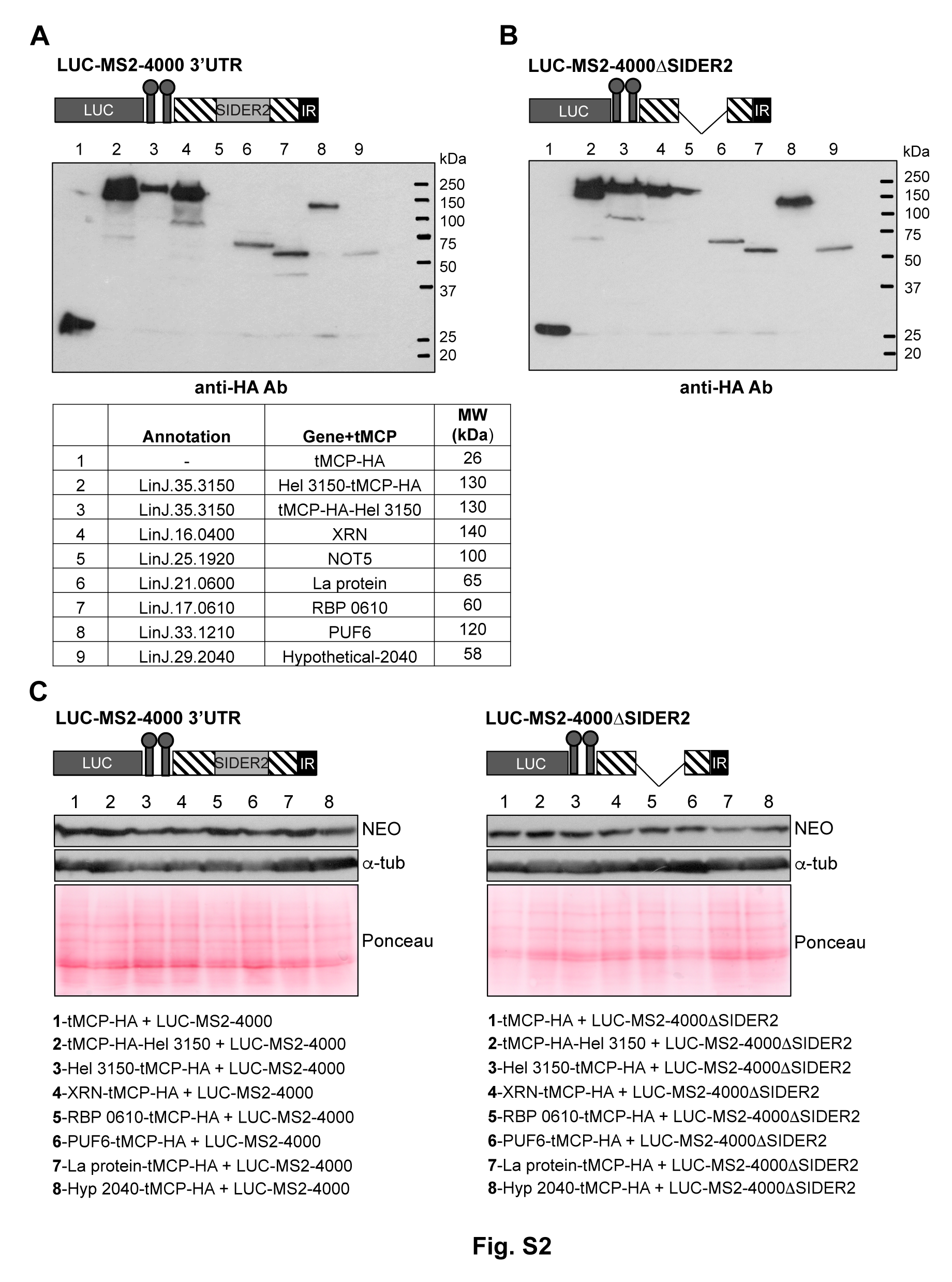

Supplement: Supplemental Material [file supp_062950.117_Supplemental_Fig_S2.tif]

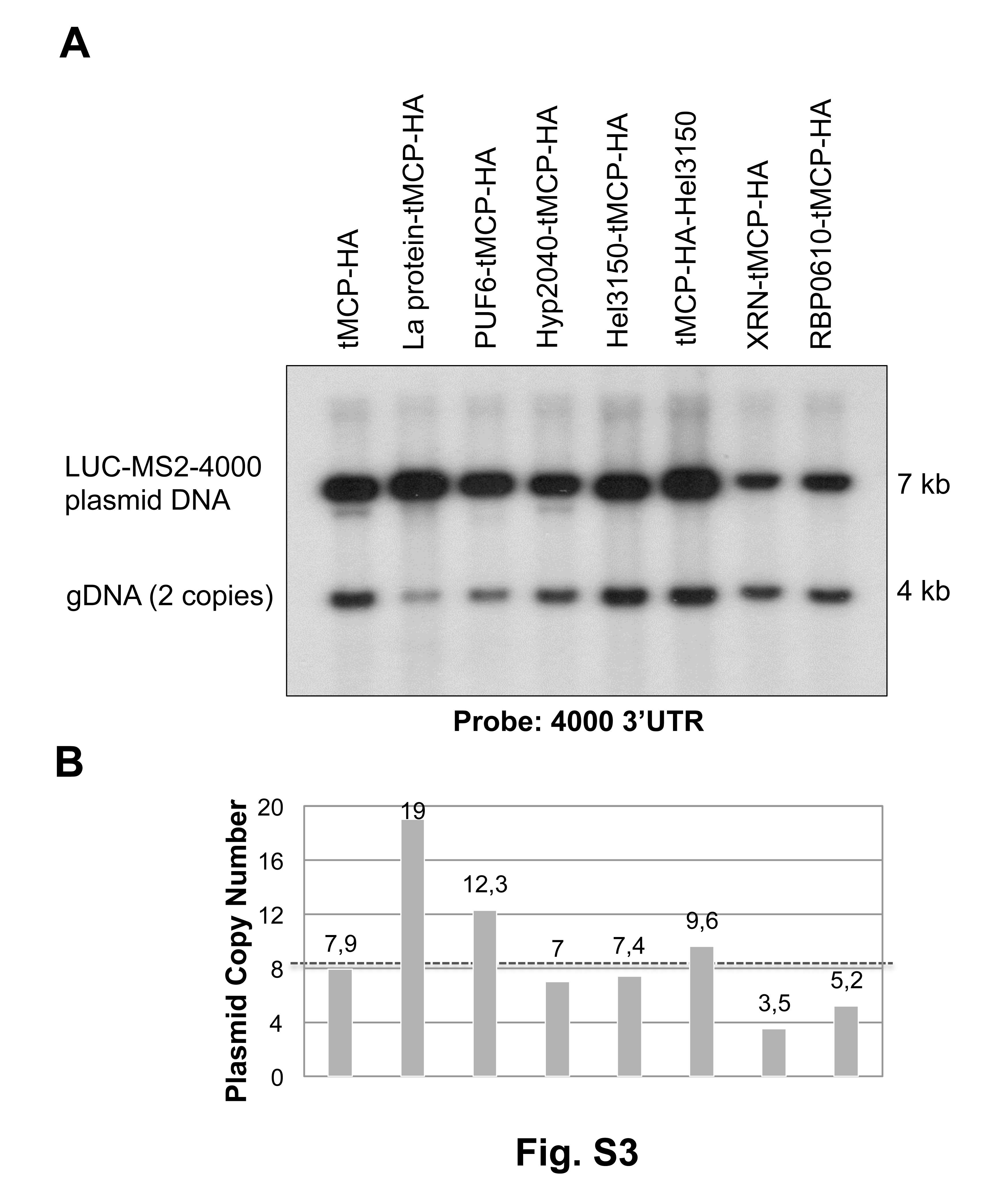

Supplement: Supplemental Material [file supp_062950.117_Supplemental_Fig_S3.tif]

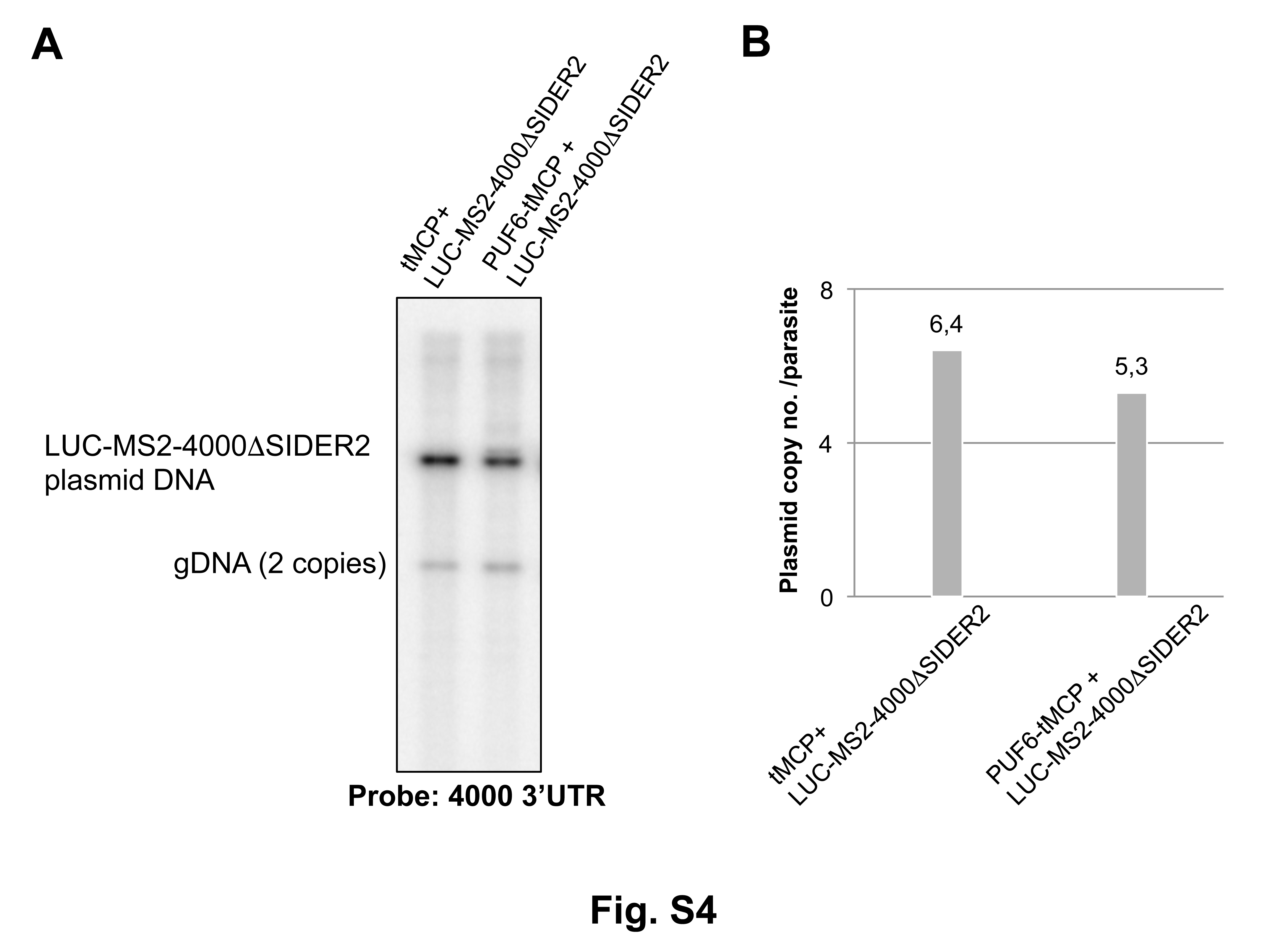

Supplement: Supplemental Material [file supp_062950.117_Supplemental_Fig_S4.tif]

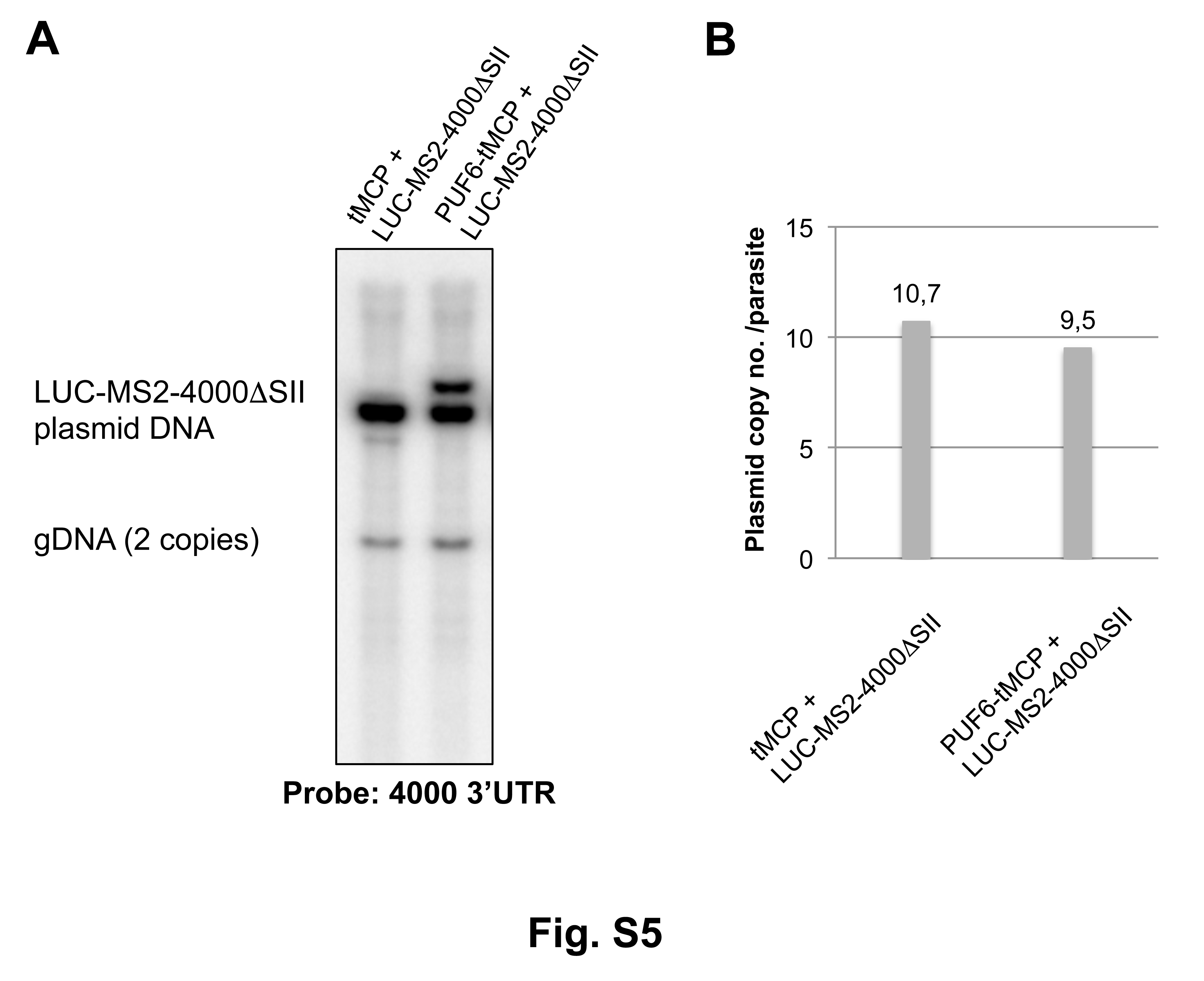

Supplement: Supplemental Material [file supp_062950.117_Supplemental_Fig_S5.tif]

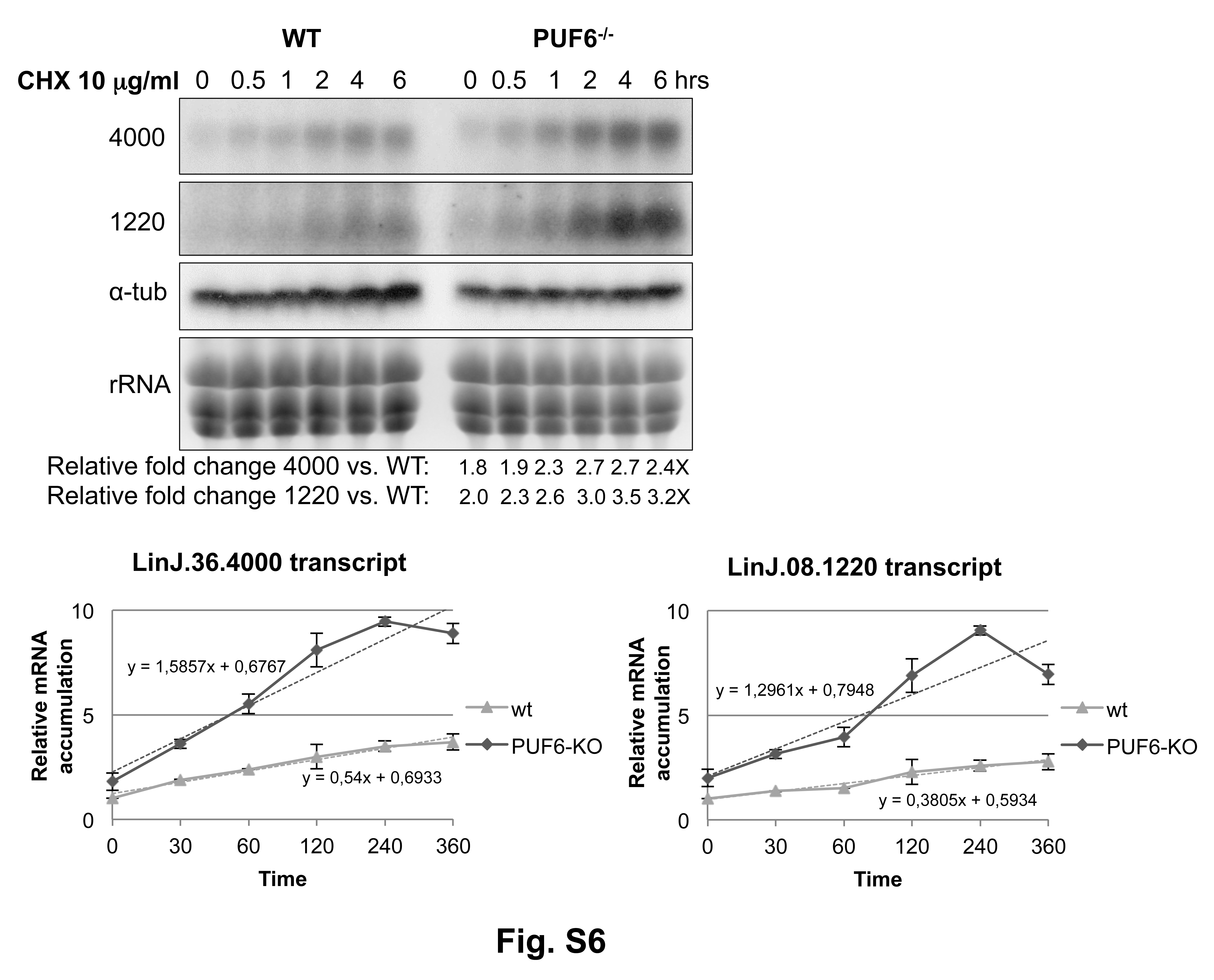

Supplement: Supplemental Material [file supp_062950.117_Supplemental_Fig_S6.tif]

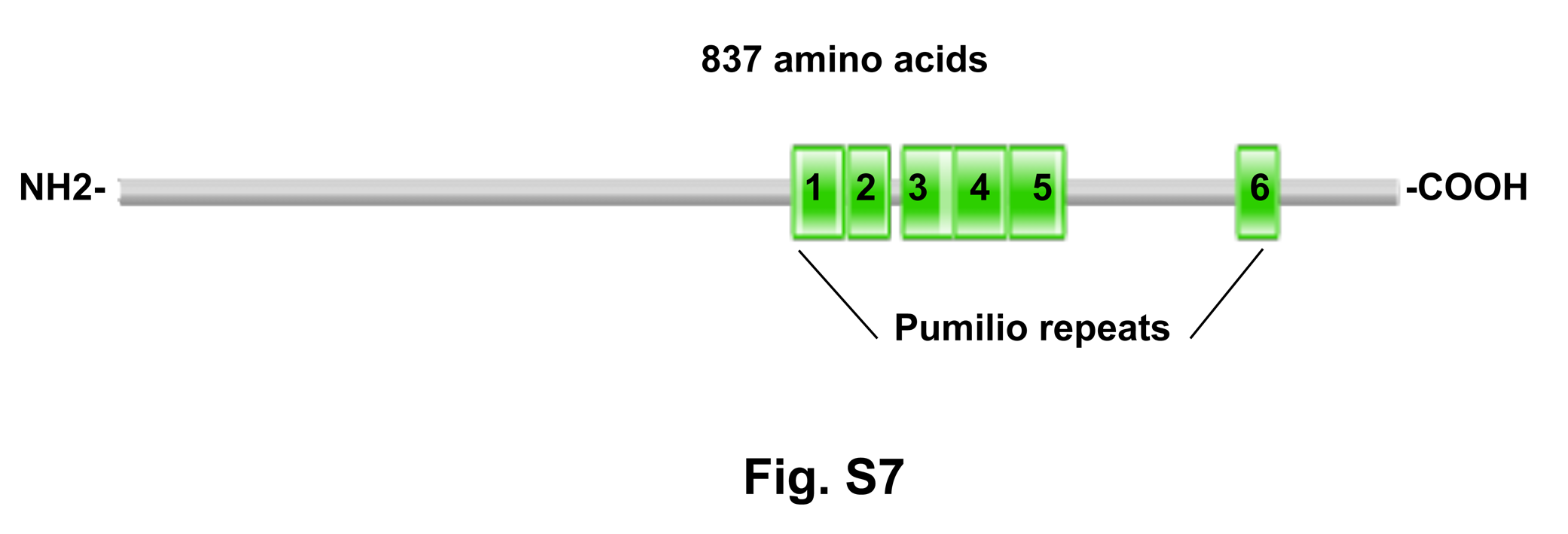

Supplement: Supplemental Material [file supp_062950.117_Supplemental_Fig_S7.tif]

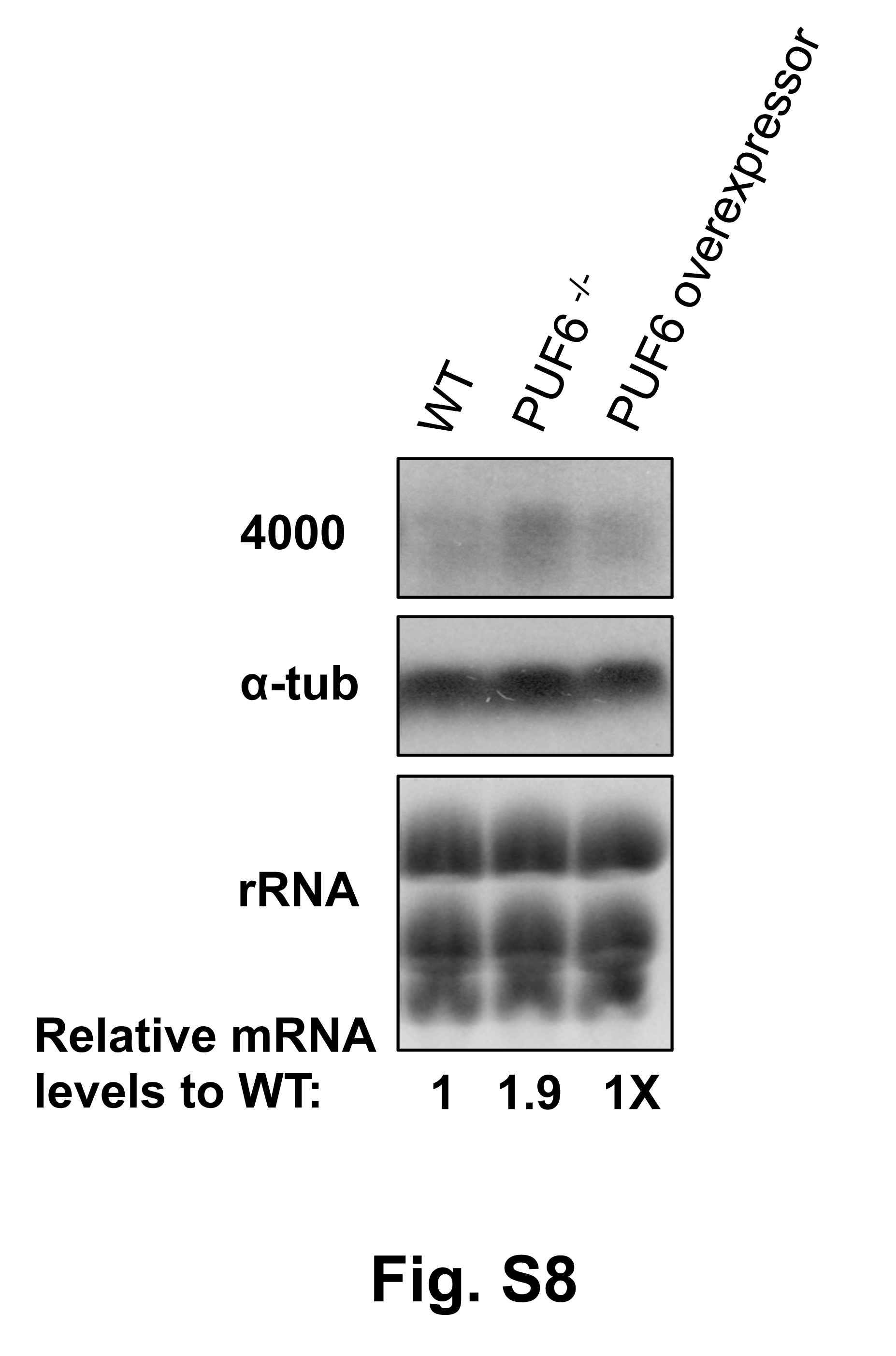

Supplement: Supplemental Material [file supp_062950.117_Supplemental_Fig_S8.tif]
