# Supplementary material for: The Pumilio-domain protein PUF6 contributes to SIDER2 retroposon-mediated mRNA decay in Leishmania
Source: RNA. 2017 Dec;23(12):1874–85. doi: 10.1261/rna.062950.117 (PMC5689007; doi:10.1261/rna.062950.117)
Supplement: Supplemental Material [file supp_062950.117_Supplemental_Information.docx]

**Supplemental Material**

**Supplemental Table S1.** List of primers used in this study.

| **Primers to generate vector pSP72αZEOα-tMCP-HA** | |
| --- | --- |
| Primer | Sequence |
| tMCP-XbaI-F | 5'GCTCTAGACCTAGGCTTAAGGCTAGCACTAGTGATGGGAAACTAGCCGTTAAC 3' |
| HA-HindIII-R | 5' CCCAAGCTTTTAAGCGTAGTCTGGCACGTCGTAAGGGTA 3' |
| **Primers to amplify candidate genes for tethering constructs** | |
| LinJ.35.3150-AvrII-F | 5' CCCCTAGGATGGAATATATCAACGATAAGAAAATTTC 3' |
| LinJ.35.3150-NheI-R | 5' CTAGCTAGCGAAGCCACCGTCGTCCACAC 3' |
| LinJ.25.1920-AvrII-F | 5' CCCCTAGGATGTCGAACATCAAGAAGGTGCA 3' |
| LinJ.25.1920-AvrII-R | 5' CCCCTAGGGTGGAGTTCATTCTCAAGGT 3' |
| LinJ.29.2040-AvrII-F | 5' CCCCTAGGATGAGTAGCACCGCTGTGGCGA 3' |
| LinJ.29.2040-NheI-R | 5' CTAGCTAGCCTCGTCGCGCCCTCGCTTCA 3' |
| LinJ.33.1210-AvrII-F | 5' CCCCTAGGATGTACTCGGAACAGAGCTGGA 3' |
| LinJ.33.1210-AvrII-R | 5' CCCCTAGGGCGGCGGTGGTGGTTGGCGCGGCCCT 3' |
| LinJ.17.0610-AvrII-F | 5' CCCCTAGGATGGACGGCCGACTTGTGCA 3' |
| LinJ.17.0610-NheI-R | 5' CTAGCTAGCAACACCCATCTGGATTGCTA 3' |
| LinJ.21.0600-AvrII-F | 5' CCCCTAGGATGTCCGACCTCGCCGATAAGA 3' |
| LinJ.21.0600-NheI-R | 5' CTAGCTAGCGTGGCGACCACCGCCACGGC 3' |
| LinJ.16.0400-XbaI-F | 5' GCTCTAGAATGGGGCTTTTAGGGCTTCGCA 3' |
| LinJ.16.0400-HindIII-R | 5' CCCAAGCTTGTACTGCACCCTCCTCCGCT 3' |
| **Primers to generate vector pSP72αNEOα-PUF6-HA for genomic integration** | |
| LinJ.33.1210-5’UTR-BglII-HindIII | 5' GAAGATCTAAGCTTCGTTCTCTCTCTGTTTTCCTCT 3' |
| LinJ.33.1210-5’UTR-SacI-R | 5' CGAGCTCTTCGATCGTCTTGCAATGTGGA 3' |
| LinJ.33.1210-ORF-XbaI-F | 5' GCTCTAGAATGTACTCGGAACAGAGCTGGA 3' |
| LinJ.33.1210-ORF-tMCP-HA-R | 5'ACGGGTCCTAACTTTGTTGCTTTAAGCGTAGTCTGGCACGTCGTAAGGGTAAGCGTAGTCTGGCACGTCGTAAGGGTAGCGGCGGTGGTGGTTGGC 3' |
| LinJ.33.1210-3’UTR-F | 5' AGCAACAAAGTTAGGACCCGT 3' |
| LinJ.33.1210-3’UTR-HindIII-R | 5' CCCAAGCTTCAGGAGGGGAAAGAAGAGAAA 3' |
| **Primers to generate a *PUF6* gene knockout** | |
| LinJ.33.1210-5’UTR-BglII-HindIII | 5' GAAGATCTAAGCTTCGTTCTCTCTCTGTTTTCCTCT 3' |
| LinJ.33.1210-DKO-Cloning-R1 | ACGGGTCCTAACTTTGTTGCTTCTAGAGGATCCTTCGATCGTCTTGCAATGTGGA |
| LinJ.33.1210-3’UTR-F | 5' AGCAACAAAGTTAGGACCCGT 3' |
| LinJ.33.1210-3’UTR-HindIII-R | 5' CCCAAGCTTCAGGAGGGGAAAGAAGAGAAA 3' |
| NEO-BamHI-F | 5' CGGGATCCATGATTGAACAAGATGGATTG 3' |
| NEO-XbaI-R | 5' GCTCTAGATCAGAAGAACTCGTCAAGAAGGC 3' |
| HYG-BamHI-F | 5' CGGGATCCATGAAAAAGCCTGAACTCACC 3' |
| HYG-XbaI-R | 5' GCTCTAGACTATTCCTTTGCCCTCGGACGA 3' |
| **Primers to create plasmids containing MS2 binding sites** | |
| 2MS2-BamHI-LUC-F | 5'CGGGATCCACATGAGGATCACCCATGTGGTTTTTGGACATGAGGATCACCCATGTCCTTTTTATGGAAGACGCCAAAAACATA 3' |
| 2MS2-BamHI-4000UTR | 5'CGGGATCCACATGAGGATCACCCATGTGGTTTTT**GG**ACATGAGGATCACCCATGT**CC**AACGGCCGTGCTCCGACAAATACA 3' |

**Supplemental Figure Legends**

**Figure S1. Western blot analysis to confirm enrichment of immunoprecipitated tandem MCP-HA (tMCP-HA) in *L. infantum* cells co-transfected with PTP-tMCP-HA and LUC-MS2-4000 3ʹUTR or MS2-LUC-4000 3ʹUTR.** Following co-immunoprecipitation (IP) against PTP-tMCP-HA using HA-magnetic beads, 0.1 volume of the IPs was subjected to Western blotting along with a volume of total lysates (TL) corresponding to approximately 2X10^6^ parasites as control. An anti-HA antibody was used for Western blots at 1:2000 dilution. The recombinant parasites expressing only PTP-tMCP-HA were used as control to eliminate unspecific interactions with tagged MCP protein. The 45 kDa protein band indicates selective enrichment of the PTP-tMCP-HA protein in IPs.

**Figure S2. Western blot analysis to confirm expression of candidate proteins identified upon tethering of tMCP to the SIDER2-containing LinJ.36.4000 3ʹUTR.** Candidate proteins were fused to tMCP-HA and co-expressed in *L. infantum* LUC-MS2-4000 3’UTR or *L. infantum* LUC-MS2-4000ΔSIDER2. (A) Western blot analysis of total lysates from *L. infantum* cells co-expressing each one of the candidate proteins (2-9, see table below) fused to tMCP-HA together with LUC-MS2-4000 3ʹUTR to assess protein expression prior to tethering assays. An anti-HA antibody at 1:2000 dilution was used for Western blotting. Estimated molecular weight of the fusion proteins is also shown. (B) Western blotting as in A but using total lysates from *L. infantum* cells co-expressing each one of the candidate proteins fused to tMCP-HA and LUC-MS2-4000ΔSIDER2. (C) Western blot analysis of total lysates from *L. infantum* co-expressing each one of the candidate proteins together with LUC-MS2-4000 3ʹUTR or LUC-MS2-4000ΔSIDER2 (as indicated in the table below) using anti-NEO and anti-α-tubulin antibodies as protein loading controls. A Ponceau staining is also included to evaluate protein loading.

**Figure S3. Plasmid copy number estimates in parasites co-expressing LUC-MS2-4000 3ʹUTR or LUC-MS2-4000ΔSIDER2 and candidate proteins fused to tandem MCP.** (A) Southern blot hybridization of total DNA (digested with NdeI) isolated from LUC-MS2-4000 3ʹUTR and tMCP-HA co-transfected parasites with a radiolabeled DNA probe corresponding to the first 1000 nucleotides of the LinJ.36.4000 3ʹUTR and recognizing both the plasmid and genomic (gDNA) DNAs. (B) Plasmid copy number estimates in each co-transfected *L. infantum* strain. The ratio of the plasmid DNA hybridization signal vs. the genomic DNA hybridization signal (2 copies; diploid genome) determines plasmid copy number per recombinant parasite, as displayed in the diagram. The intensity of the hybridization signals (plasmid DNA vs. gDNA) was measured by PhosphorImager. The values on the top of the graph indicate the plasmid copy number per diploid genome in each double transfectant.

**Figure S4. Plasmid copy number estimates in parasites co-expressing LUC-MS2-4000ΔSIDER2 and tMCP or PUF6-tMCP.** (A) Southern blot hybridization of total DNA isolated from co-transfected parasites as indicated in Figure S3A. (B) Plasmid copy number in each co-transfected *L. infantum* strain was estimated as in Figure S3B. The intensity of the hybridization signals was measured by PhosphorImager and the values on the top of the graph indicate the plasmid copy number per diploid genome in each double transfectant.

**Figure S5. Plasmid copy number estimates in parasites co-expressing LUC-MS2-4000ΔSII and tMCP or PUF6-tMCP.** (A) Southern blot hybridization of total DNA isolated from co-transfected parasites as indicated in Figure S3A. (B) Plasmid copy number in each co-transfected *L. infantum* strain was estimated as in Figure S3B. The intensity of the hybridization signals was measured by PhosphorImager and the values on the top of the graph indicate the plasmid copy number per diploid genome in each double transfectant.

**Figure S6. Global inhibition of translation elongation by cycloheximide (CHX) increases accumulation of the LinJ.36.4000 and LinJ.08.1220 endogenous transcripts in PUF6^-/-^ parasites.** Here, we compared mRNA accumulation between wild type (WT) parasites and the PUF6^-/-^ knockout strain. CHX treatment was carried out in a time course manner up to 6 h post-treatment. Total mRNAs were isolated and subjected to northern blotting. The blots were first hybridized with a radiolabeled probe specific to the 4000 open reading frame (ORF) and subsequently with a probe recognizing the 1220 ORF. Loading variations were normalized based on hybridization signal intensities of the α-tubulin mRNA and the fold differences are shown below the blots. The charts shown in the lower panels reflect the normalized values for each transcript.

**Figure S7. Predicted Pumilio domains in the *Leishmania* PUF6 homolog.** Using Pfam search engine (pfam.xfam.org), the protein sequence of *L. infantum* LinJ.33.1210 was analyzed for known protein domains. The result, which is based on the presence of conserved domains among annotated proteins in available databases (Pfam), indicates that the *Leishmania* PUF6 possesses 6 predicted Pumilio repeats, which are depicted in the schematic drawing numbered by 1-6.

**Figure S8.** **PUF6 overexpression does not affect steady state levels of SIDER2-containing mRNAs**. In order to ascertain that increased destabilization of SIDER2-harboring mRNAs upon tethering of PUF6 is not due to PUF6 overexpression, a plasmid expressing PUF6-HA was generated and transfected into *L. infantum* and the expression of endogenous 4000 transcript was measured by northern blot hybridization. The α-tubulin signal used to correct for loading variations and the signal intensities were calculated by Image Quant software. The values indicated at the bottom of the blot represent the normalized values relative to wild type (WT). The result presented here is representative of two independent experiments yielding consistent data.
